# Systematic identification of the key candidate genes in breast cancer stroma

**DOI:** 10.1186/s11658-018-0110-4

**Published:** 2018-09-17

**Authors:** Yanxia Wang, Hui Xu, Baoan Zhu, Zhenling Qiu, Zaijun Lin

**Affiliations:** 1 Department of Emergency Medicine, Shanghai Ninth People’s Hospital, Shanghai Jiao Tong University School of Medicine, Center for Specialty Strategy Research of Shanghai Jiao Tong University China Hospital, Shanghai, 200000 People’s Republic of China; 2 Department of Biochemistry, Luohe Medical College, Luohe, 462002 Henan Province People’s Republic of China; 3 Shandong Yantai Laiyang Center Hospital, 111 Changshan Road, Laiyang, 265200 Shandong Province China; 4 The Orthopedic Department of Shanghai Hospital of Traditional Chinese Medicine, 274 Zhijiang Middle Road, Jing’an District, Shanghai, 200000 People’s Republic of China

**Keywords:** Differentially expressed genes, *COL1A1*, *FN1*, Breast cancer

## Abstract

**Background:**

Tumor microenvironment, in particular the stroma, plays an important role in breast cancer cell invasion and metastasis. Investigation of the molecular characteristics of breast cancer stroma may reveal targets for future study.

**Methods:**

The transcriptome profiles of breast cancer stroma and normal breast stroma were compared to identify differentially expressed genes (DEGs). The method was analysis of GSE26910 and GSE10797 datasets. Common DEGs were identified and then analyses of enriched pathways and hub genes were performed.

**Results:**

A total of 146 DEGs were common to GSE26910 and GSE10797. The enriched pathways were associated with “extracellular matrix (ECM) organization”, “ECM-receptor interaction” and “focal adhesion”. Network analysis identified six key genes, including *JUN*, *FOS*, *ATF3*, *STAT1*, *COL1A1* and *FN1*. Notably, *COL1A1* and *FN1* were identified for the first time as cancer stromal key genes associated with breast cancer invasion and metastasis. Oncome analysis showed that the high expression levels of *COL1A1* and *FN1* correlated to an advanced stage of breast cancer and poor clinical outcomes.

**Conclusions:**

We found that several conserved tumor stromal genes might regulate breast cancer invasion through ECM remodeling. The clinical outcome analyses of *COL1A1* and *FN1* suggest these two genes are promising targets for future studies.

## Introduction

Breast cancer develops from primary solid tumors that invade locally. Subsequently, distant metastasis occurs. It is this distant impact that causes more than 90% of cancer-related deaths [1].

The normal mammary gland is comprised of a central lumen, a well-defined layer of epithelial cells, a layer of contractile myoepithelial cells, basement membrane and the interstitial matrix or the stroma, which consists of randomly organized fibrillar collagen [2]. As the tumor microenvironment is known to affect tumor invasion and metastasis, it is important to consider how these elements interplay with tumors. Within the tumor microenvironment, stroma, which contain fibroblasts and immune cells are often altered as cancer progresses. Alterations include the activation of the fibroblasts, the remodeling of the extracellular matrix (ECM) and angiogenesis [3, 4].

The ECM regulates breast development and differentiation. It also provides structural support for the cells and mediates epithelial–stromal communication. It undergoes constant remodeling during normal mammary development, and an imbalance in this process causes ECM dysregulation and disorganization, further resulting in breast cancer [2]. However, the balance between the deposition of ECM components and their concomitant degradation by matrix metalloproteinases (MMPs) is closely associated with tumor growth and invasion [5]. ECM dynamics reveal that deposition and cross-linking of collagen lead to increased collagen fiber linearization and thickening. The orientation of the collagen fibers is also profoundly altered: they align perpendicular to the tumor boundary, forming migration tracks for invasive tumor cells to exit the tumor tissue and enter the blood stream [2].

During this process, stromal composition is also altered. Cancer-associated fibroblasts and immune cells appear and participate in the activation of local macrophages, fibroblasts and endothelial cells and the recruitment of a variety of leukocyte subsets [6, 7]. The activated macrophages and recruited leukocytes secrete their own repertoire of cytokines, chemokines and proteolytic enzymes, and then ECM degradation occurs.

The whole process facilitates the infiltration of invasive tumor cells through the basement membrane ECM into the surrounding breast stroma from the established migration tracks [8, 9]. Therefore, alterations in the activation of fibroblasts, remodeling of the extracellular matrix (ECM) and angiogenesis in cancer stroma can be considered as parts of the tumor invasion processes. In addition, the molecular markers regulating these cancer stromal alteration processes are important.

ECM deposition and degradation also play critical roles in breast cancer and its metastasis. ECM component degradation is primarily induced by MMPs, which are classified in six groups: collagenases, gelatinases, stromelysins, matrilysins, membrane-type MMPs, and other non-classified MMPs [10]. Importantly, the members of the MMP family are significantly associated with tumor progression. For example, MMP-2 and MMP-9 regulate the degradation of collagen type IV, a major element of the basement membrane [11]. MMP-1, MMP-2, MMP-9 and MMP-14 promote breast cancer growth and metastasis [12–15]. Some MMPs, including MMP-1, MMP-7 and MMP-12, have also been reported as markers associated with poor prognosis in breast cancer [16].

Lysyl oxidase (LOX), an amine oxidase enzyme, catalyzes cross-linking between collagens and elastins in the ECM. It increases ECM stiffness, thereby promoting invasive behavior in breast cancer cells [11]. Experiments in vitro showed that LOX overexpression promotes breast cancer invasion, metastasis and epithelial-to-mesenchymal transition (EMT) [17]. Silencing LOX suppresses breast cancer metastasis [18].

Besides MMPs and LOX, other ECM modifying enzymes, such as urokinase plasminogen activator (uPA) system and cathepsins, are also associated with breast cancer and metastatic progression. Additionally, matricellular proteins, a group of ECM glycoproteins, are greatly upregulated during tissue remodeling, e.g., during mammary gland involution and pathological conditions such as cancer [19]. Although these matrix proteins may have a limited effect on the mechanical structure of the ECM, they are actually important for cell regulators and the modulation of signaling pathways [20]. Proteins of the matricellular family include tenascin C (TNC), osteopontin/secreted phosphoprotein 1 (SPP1), periostin (POSTN), acidic and rich in cysteine (SPARC) and thrombospondin (THBS). They play important roles in breast cancer and metastasis.

The roles of proteins related to ECM remodeling and cell invasion have been studied well. However, the context-dependent functions of these genes and pathways in the tumor microenvironment are unclear.

The stromal tissue within the tumor microenvironment includes fibroblasts, adipocytes, and blood and lymph vessels. Alterations in these components influence tumor progression [9]. Here, we investigated the molecular characteristics of the tumor stroma, and identified the biomarker candidates related to breast cancer progression and metastasis. We discuss their potential roles in early tumor detection and tumor therapy. We analyzed the GSE26910 and GSE10797 datasets and compared the transcriptome profiles of stroma surrounding invasive breast primary tumors and normal breast stroma. We then overlapped the differentially expressed genes (DEGs) from the comparisons to identify commonality. The common DEGs were functionally enriched, and a protein–protein interaction (PPI) network was constructed to identify the key cancer stroma genes. Our results reveal the dysregulated stromal genes in invasive breast cancer and may provide novel targets for therapy against breast cancer invasion and metastasis.

## Materials and methods

### Derivation of genetic data

The gene expression profile datasets GSE26910 and GSE10797 were downloaded from the Gene Expression Omnibus (GEO) database (http://www.ncbi.nlm.nih.gov/geo), repository of gene expression data from high-throughput hybridization arrays, ChIPs and microarrays. GSE26910 contains six samples of stroma surrounding invasive breast primary tumors and six matched samples of normal stroma obtained using the platform GPL570 Affymetrix Human Genome U133 Plus 2.0 Array. GSE10797 contains 28 samples of invasive breast cancer stroma and five samples of normal stroma obtained using GPL571 Affymetrix Human Genome U133A 2.0 Array.

### Data processing

Series matrix file(s) (SMF) and an annotation soft table were collected from GSE26910 and GSE10797. The expressions of genes in each sample from were extracted from the SMF. R3.2.2 software was used to pre-process the downloaded raw data via background correction and quantile normalization. Using Perl [21], probes were transformed into gene names. For genes corresponding to more than one probe, gene expression levels were determined using the average probe values. An “impute” package [22] was applied to complement missing expression with its adjacent value.

### Screening of DEGs

We performed two comparisons: one between breast cancer stroma and normal stroma from GSE26910; and the other between those tissue types from GSE10797. Limma package [23] was used to screen DEGs with *p* < 0.05 and |log (fold change) | ≥ 1. The DEGs were grouped into upregulated and downregulated genes, and cluster software was used to cluster the samples based on their gene expression values. In addition, the samples were subtyped based on clustering analysis and heat maps between the gene expression values and samples were generated. A volcano plot displaying the log-fold change against the –log(10) (*p*-value) was also generated using all the genes that were different in invasive breast cancer stroma. All DGEs from the GSE26910 dataset were matched with the DEGs in GSE10797 dataset using DIDS algorithm to identify the common DEGs consistently downregulated or upregulated in the two microarrays.

### Functional annotation and pathway enrichment analysis

To functionally annotate the common DEGs, R packages, such as GOstats and clusterProfiler [24], were used to analyze the significantly enriched Gene Ontology (GO) biological processes and Kyoto Encyclopedia of Genes and Genomes (KEGG) pathways (*p* < 0.05).

### Protein–protein interaction network construction

PPI databases from HPRD [25], BIOGRID [26] and PIP [27] were downloaded to extracted 562,252 pair interactions. The common DEGs identified above were directly mapped to the PPI databases to acquire significant PPI pairs from a range of sources, including data from experimental studies and data retrieved by text mining and homology searches. Cytoscape 3.2.1 [28] was used to construct the interaction network by importing the interacted gene pairs into our curated PPI database. Large nodes could then be identified based on the degree for each node.

### Oncomine analysis

The expression levels of select genes in invasive breast cancer were analyzed using Oncomine gene expression array datasets (www.oncomine.org), which is an online cancer microarray database designed to facilitate discovery from genome-wide expression analyses. In this study, associations of mRNA expression levels with invasive breast tumor presence, advanced stage, recurrence and metastatic events were analyzed using Student’s *t*-test to generate the *p* value. The *p* value was set up at 0.05 and fold change was defined as 1.5. In many instances, we found several significant correlations in each clinical event, but only showed one or two representative examples.

## Results

### Differentially expressed genes between stroma surrounding invasive breast primary tumors and normal breast stroma cells

**Fig. 1:**
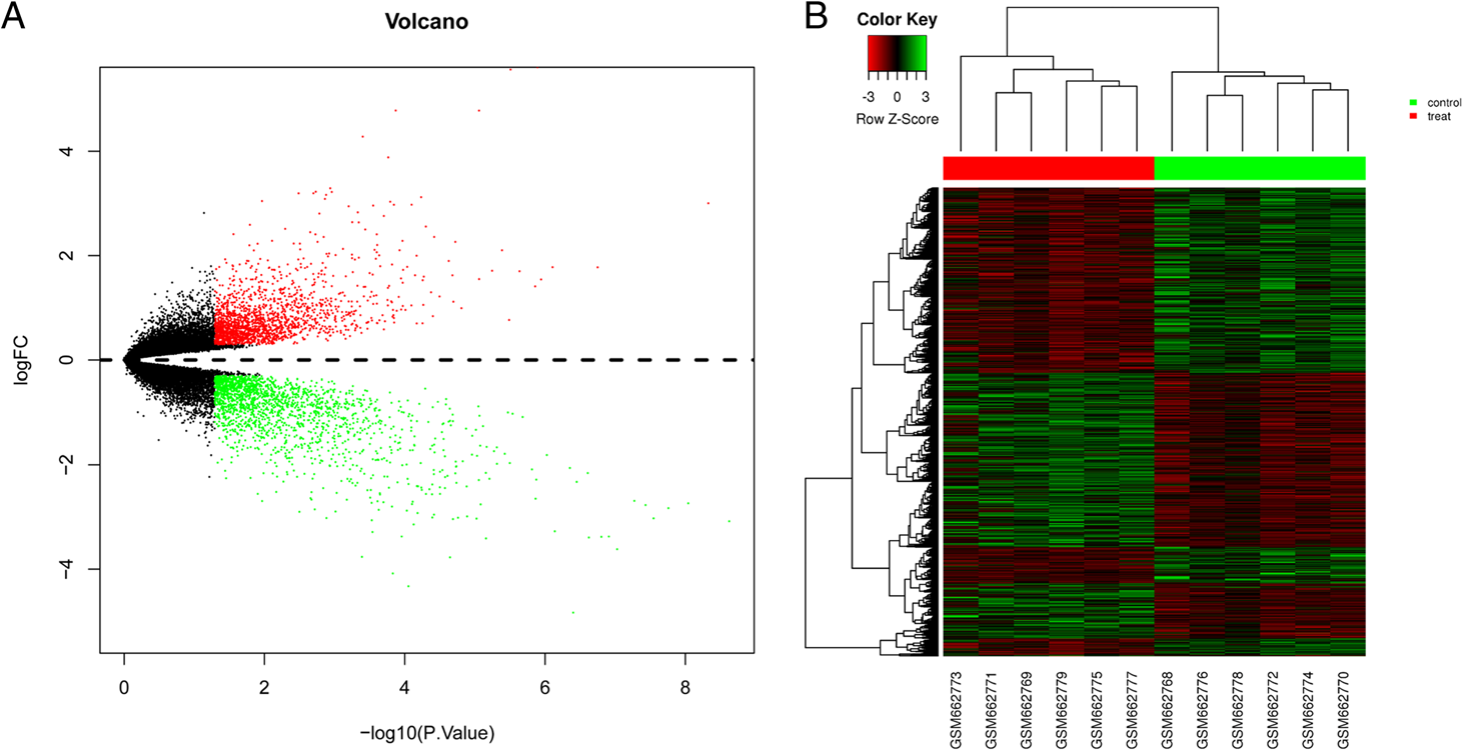
Heat map and volcano plot for differentially expressed genes (DEGs) in breast cancer stroma cells compared with normal breast stroma from GSE26910. **a** – Volcano plot of DEGs in breast cancer stroma cells from GSE26910. x-axis: LogFC, large magnitude fold changes; y-axis: -log10 of *p*-value, high statistical significance. Red and green points: log_2_|fold change| ≥ 1 and *p* value < 0.05. Black points: log_2_|fold change| < 1 or *p* value > 0.05. **b** – Dendrogram of DEGs identified via cluster analysis. Each column represents one sample. The green header indicates normal stromal cells and red indicates breast cancer stromal cells. Each row represents one gene, with red representing high relative expression, and green representing low relative expression

**Fig. 2:**
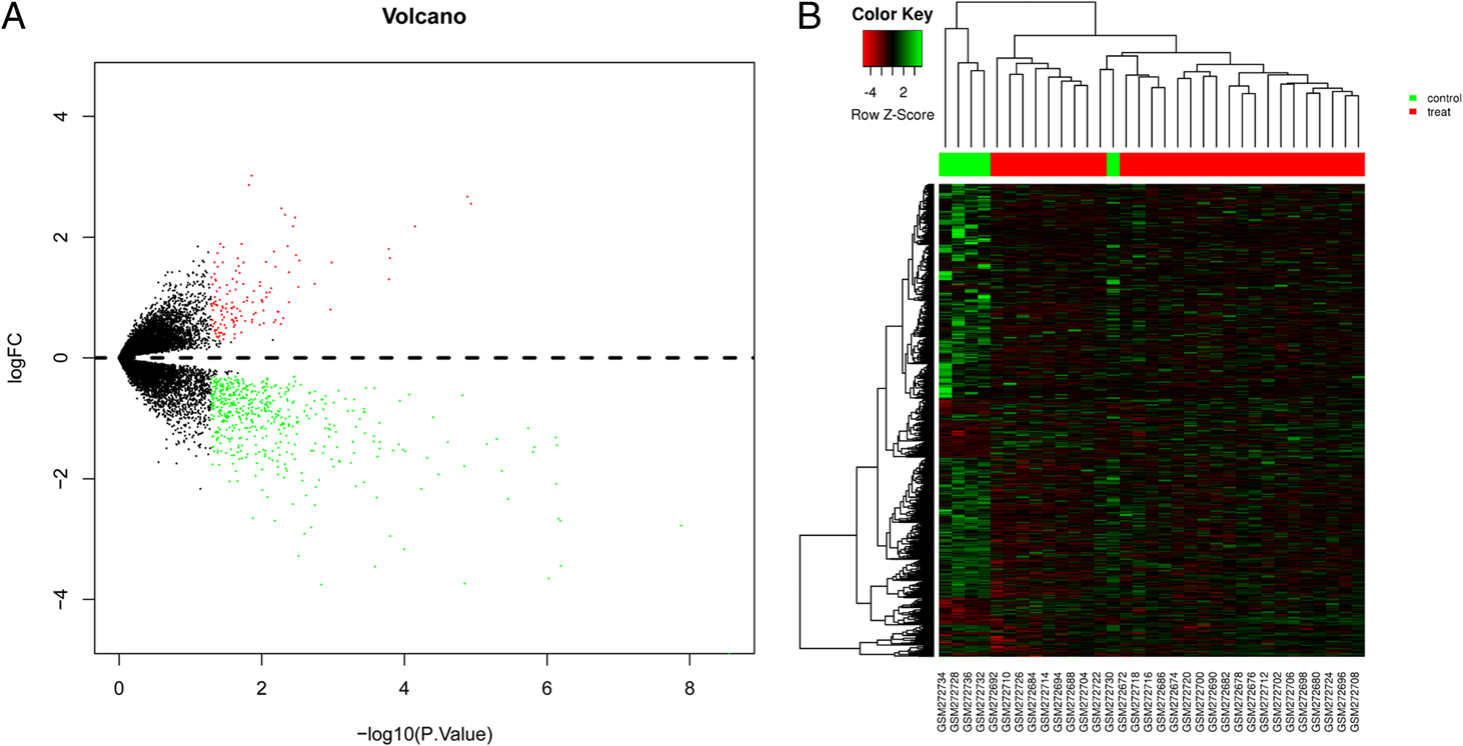
Heat map and volcano plot for differentially expressed genes (DEGs) in breast cancer stroma cells compared with normal breast stroma from GSE10797. **a** – Volcano plot of DEGs in breast cancer stroma cells from GSE10797. x-axis: LogFC, large magnitude fold changes; y-axis: -log10 of *p*-value, high statistical significance. Red and green points: log_2_|fold change| ≥ 1 and *p* value < 0.05. Black points: log_2_|fold change| < 1 or *p* value > 0.05. **b** – Dendrogram of DEGs identified via cluster analysis. Each column represents one sample. The green header indicates normal stromal cells and red indicates breast cancer stromal cells. Each row represents one gene, with red representing high relative expression, and green representing low relative expression

The GSE26910 and GSE10797 databases were downloaded and analyzed. First, we analyzed the gene expression profiles for stroma surrounding invasive breast primary tumors and for normal breast stroma cells. A total of 3708 DEGs were identified from the GSE26910 dataset (Fig. 1) and 665 DEGs from GSE10797 dataset (Fig. 2). Second, to identify common genes between these two datasets, we overlapped the DEGs in the two comparisons, revealing 146 common DEGs with consistent changes (Table 1).

**Table 1:** The common genes altered in both the GSE26910 and GSE10797 datasets

Overlapping gene	logFC (GSE26910)	logFC (GSE10797)	Overlapping gene	logFC (GSE26910)	logFC (GSE10797)
FOSB	−3.29	−1.57	CNTRL	− 0.74	− 0.66
CLDN5	−3.27	− 0.94	AV720803	− 0.74	− 1.31
IL33	−3.14	−1.06	DYNC2LI1	−0.72	− 0.71
CD36	−3.02	−0.82	CITED1	−0.71	−1.70
CFD	−3.02	−1.87	CD59	−0.69	−1.25
TGFBR3	−2.99	−1.22	TSPAN14	−0.65	−0.88
SEMA3G	−2.92	−0.95	SPTBN1	−0.65	− 0.34
FAM189A2	−2.83	−1.57	CNKSR2	−0.65	−1.04
ZBTB16	−2.80	−0.51	SEPP1	− 0.65	−1.16
RELN	−2.72	−1.29	IFNGR1	− 0.61	−0.75
APOD	−2.67	−2.04	MBNL2	−0.60	− 0.66
PPL	−2.56	−1.39	GLTSCR1L	−0.59	− 0.95
DLK1	−2.52	−0.56	EXOSC10	−0.59	−1.55
SYNM	−2.43	−2.95	DST	−0.59	− 0.87
FOS	−2.40	−0.91	U2SURP	−0.55	−1.09
SPRY2	−2.17	−1.40	SNORD23	−0.55	−1.32
ITIH5	−2.09	−0.87	MCCC1	−0.52	−1.02
AV764378	−1.96	− 1.38	ZFP36L2	−0.49	−0.42
TNXB	−1.74	−0.60	CERS1	−0.44	−0.68
JUN	−1.72	−2.08	TLE4	−0.43	−0.67
AMOTL2	−1.71	−0.61	GABARAPL3	−0.43	− 0.96
ABCA8	−1.68	−2.42	NCALD	−0.43	−1.52
DUSP6	−1.61	−1.06	RPL7	−0.40	−0.55
LHFP	−1.50	−1.93	GSN	−0.39	−0.52
PTH2R	−1.49	−0.87	EZH1	−0.37	−0.68
CLEC1A	−1.42	−0.82	COQ8A	−0.37	−1.35
CXCL1	−1.39	−0.79	RPS6	−0.37	−0.66
SFRP1	−1.37	−1.87	ERCC5	−0.36	−1.74
EGR1	−1.34	−0.60	TJP2	−0.35	−1.16
RGL1	−1.33	−0.97	SRSF4	−0.34	−0.80
FGF13	−1.28	−0.99	SUMO3	0.43	0.55
HMGN3	−1.27	−1.77	BASP1	0.50	1.01
CASQ2	−1.24	−0.50	COMMD10	0.50	1.11
COLGALT2	−1.16	−0.75	FAM89B	0.52	0.55
ACACB	−1.14	−1.22	HILPDA	0.59	0.83
OLFML2A	−1.14	−0.46	CAPZB	0.60	0.40
ENOSF1	−1.12	−0.88	FCGR2A	0.63	1.59
ABCA5	−1.10	−1.23	LEF1	0.64	0.80
GHR	−1.09	−1.47	PSMB5	0.64	1.16
ITGA6	−1.08	−0.65	CERS6	0.67	0.92
ADRB2	−1.08	−2.27	CALU	0.75	0.76
VPS37B	−1.05	−0.59	MAGED1	0.76	0.92
ATF3	−1.05	−1.43	TLL2	0.77	0.83
CCNL1	−1.04	−0.94	COL1A1	0.78	1.30
IGFBP6	−1.01	−1.28	SPARC	0.79	1.58
IER2	−1.00	−2.04	S100P	0.91	2.87
ERV3–2	−1.00	−1.16	TNFRSF12A	1.03	0.56
ECHDC2	−0.98	−1.90	THY1	1.07	0.61
MAOA	−0.97	−0.44	STAT1	1.09	0.66
GNAZ	−0.95	−0.91	RAI14	1.16	0.90
CBX7	−0.95	−0.98	COL5A2	1.18	1.76
MID1	−0.93	−0.75	NID2	1.20	1.28
ANXA1	−0.93	−2.12	SPON1	1.25	1.20
PDE9A	−0.92	−1.38	POSTN	1.43	1.62
MAFF	−0.91	−1.59	VCAN	1.45	0.96
TLE2	−0.89	−0.88	COL5A1	1.55	1.65
SH3BP5	−0.89	−1.00	FN1	1.56	2.56
ICAM2	−0.89	−0.84	BGN	1.61	1.81
STAB2	−0.87	−0.46	AEBP1	1.64	1.58
MAP3K8	−0.87	−0.75	PLXDC2	1.67	1.10
CIRBP	−0.85	−1.08	FAP	1.77	1.11
TPD52L1	−0.85	−0.99	LRRC15	1.78	1.89
NFIB	−0.84	−1.11	SNX10	1.84	1.70
HDAC4	−0.84	−1.46	SMYD3	1.91	0.52
SESN1	−0.83	−0.40	ASPN	1.94	3.02
PER2	−0.82	−1.08	CDH11	1.99	0.84
LOC101929115	−0.81	−1.43	IFI30	2.23	0.62
HMGCR	−0.80	−0.57	GREM1	2.41	1.01
AUH	−0.79	−1.11	MMP1	3.05	1.84
C1orf115	−0.79	−0.48	SULF1	3.12	1.66
MIR4738	−0.79	−1.29	MATN3	3.22	1.59
CXCL2	−0.77	−2.91	COL11A1	5.56	2.33
CYP21A1P	−0.77	−2.03	COL10A1	5.60	1.52

*MMP1*, a member of the MMP family that is an inducer of ECM degradation, was highly expressed in breast cancer stroma. *POSTN* and *SPARC* respectively encode periostin and acidic and rich in cysteine, and are members of the matricellular family. They were also upregulated. These findings suggest that the dysregulated genes in invasive breast cancer stroma are involved in encoding ECM components.

### Gene ontologies for biological processes and KEGG pathway enrichment analysis

To investigate the enriched pathways of the common genes, the GO and KEGG databases were used for the pathway analyses. In the GO biological analysis, 870 processes were enriched (Table 2). “Extracellular matrix organization” is the top pathway enriched by 24 genes. These genes, including *POSTN*, *SPARC*, *COL1A1*, *COL5A1*, *COL5A2*, *COL10A1* and *COL11A1*, were also responsible for encoding collagens, which are the most abundant proteins in the ECM [29]. The significantly enriched KEGG pathways included “ECM-receptor interaction” (Table 2). Some of the genes involved in this pathway encode ECM remodeling-related proteins, such as collagens (*COL1A1*, *COL5A1*, *COL5A2*, and *COL11A1*), integrin (*ITGA6*), tenascin (*TNXB*) and fibronectin (*FN1*). In addition, the “Focal adhesion” pathway related to ECM ontologies was also enriched significantly.

**Table 2:** The significant GO biological processes and KEGG pathways enriched by the common genes identified in breast cancer stromal cells

Pathways	*p* value	Term
GOBPID		
GO:0030198	5.46E-15	Extracellular matrix organization
GO:0043062	5.78E-15	Extracellular structure organization
GO:0048523	2.14E-10	Negative regulation of cellular process
GO:0048519	8.87E-09	Negative regulation of biological process
GO:0022603	2.35E-08	Regulation of anatomical structure morphogenesis
GO:0022610	3.59E-08	Biological adhesion
GO:0009888	5.01E-08	Tissue development
GO:0007155	1.13E-07	Cell adhesion
GO:0044236	1.60E-07	Multicellular organismal metabolic process
GO:0050793	2.19E-07	Regulation of developmental process
KEGGID		
4512	3.01E-07	ECM-receptor interaction
4510	0.000316	Focal adhesion
5146	0.001049	Amoebiasis
5140	0.001126	Leishmaniasis
4380	0.002759	Osteoclast differentiation
5200	0.009171	Pathways in cancer
4974	0.012054	Protein digestion and absorption
5323	0.017842	Rheumatoid arthritis
4620	0.025932	Toll-like receptor signaling pathway
5210	0.03085	Colorectal cancer

### Identification of key genes associated with breast cancer stroma alteration

**Fig. 3:**
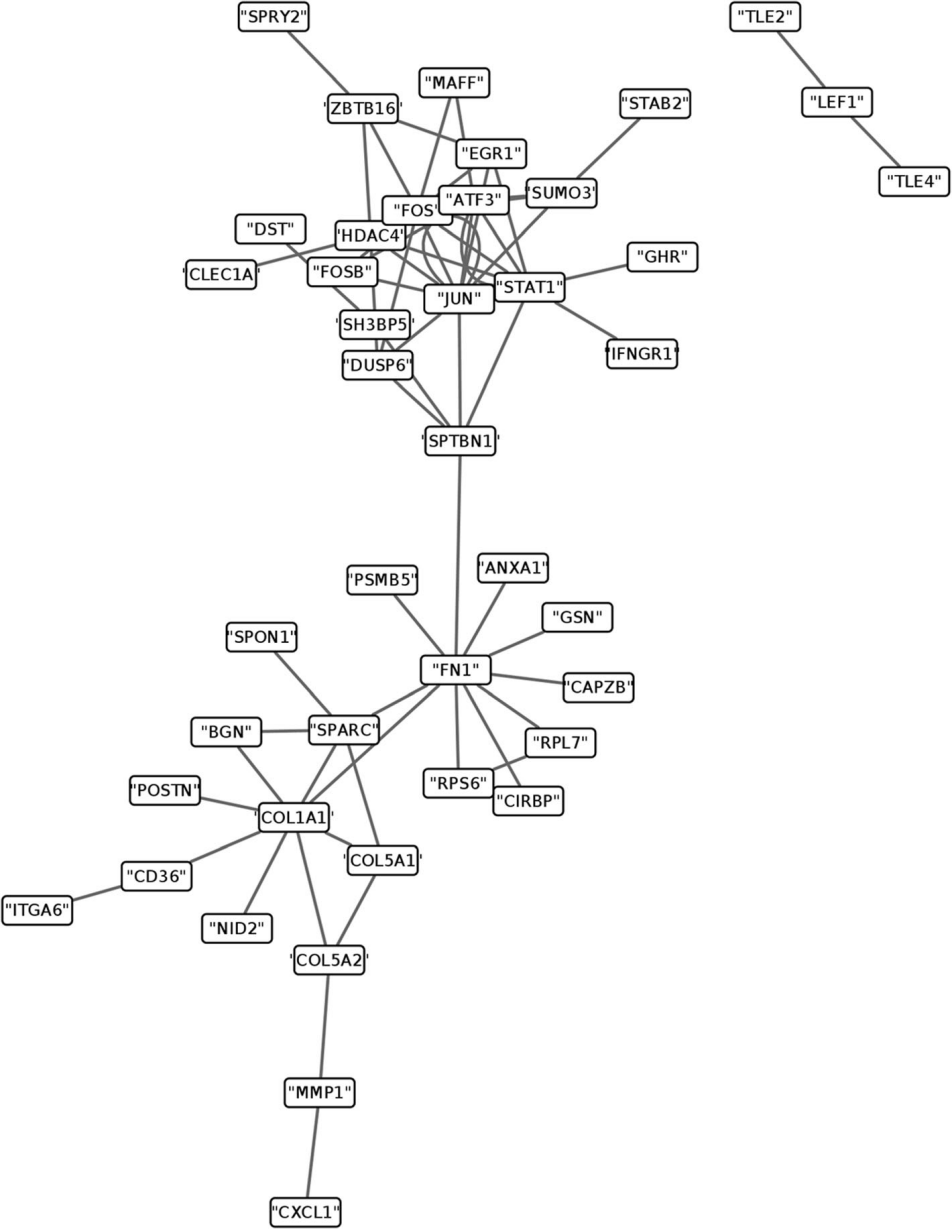
Protein–protein interaction networks of common differentially expressed genes (DEGs) consistently up- or downregulated in both comparisons

To identify key genes involved in breast cancer stroma alteration, a network analysis of the 146 common genes was conducted using PPI datasets. Proteins are constructed as nodes in protein interaction networks (PINs) and their interactions are edges [30]. The biological network results revealed 66 pair interactions (Fig. 3). Based on the degree of each node in biological network, we further identified several key genes with many interactions, including JUN, ATF3, COL1A1, FOS, STAT1 and FN1.

JUN/c-jun is a component of the transcription factor protein 1 (AP-1) complex and is phosphorylated by jun N-terminal kinase 1 (JNK1) [31], while c-jun correlates with MMP-9, which degrades ECM. Overexpression of c-jun has been reported to induce MMP-9 protein expression and activity, and elevated MMP-9 expression increases the numbers of invasive cells [32]. In breast cancer, activated c-jun is predominantly expressed at the invasive front and plays a role in proliferation and angiogenesis [31].

ATF3 (activating transcription factor), a stress response gene, has been reported to affect MMP-13 transcription. Knockdown of ATF3 expression in breast cancer cells decreases cell migration [33, 34].

Fos is a member of Fos family of AP-1 transcription factors. Like c-jun, high c-Fos protein levels promote high MMP-9 expression, whereas high FosB levels significantly correlate with MMP-1 overexpression [35]. c-Fos also enhances the invasion of the breast cancer cell line, MCF7.

STAT-1 is the first member of the family of signal transducers and activators of transcription (STATs). Knockdown of STAT1 in cancer-associated fibroblast co-cultured with human breast cancer cells altered cancer cell proliferation and delayed early breast cancer progression. This indicates a role for STAT1 as a stromal contributor of tumorigenesis [36].

Collagen 1A1 (COL1A1) belongs to the collagen family, members of which are major components of the tumor-stromal environment with important roles in cancer cell behavior [37]. Fibronectin1 (FN1) is a heterodimeric glycoprotein form at the cell surface and ECM, and is associated with cell adhesion, cell migration, wound healing and cell metastasis [38].

In conclusion, *JUN*, *FOS*, *ATF3* and *STAT1* have been reported to be associated with breast cancer invasion and directly or indirectly participate in ECM remodeling. Of note, the roles of *COL1A1* and *FN1* related to breast tumor invasion are still unclear.

### *COL1A1* and *FN1* in human breast tumors

**Fig. 4:**
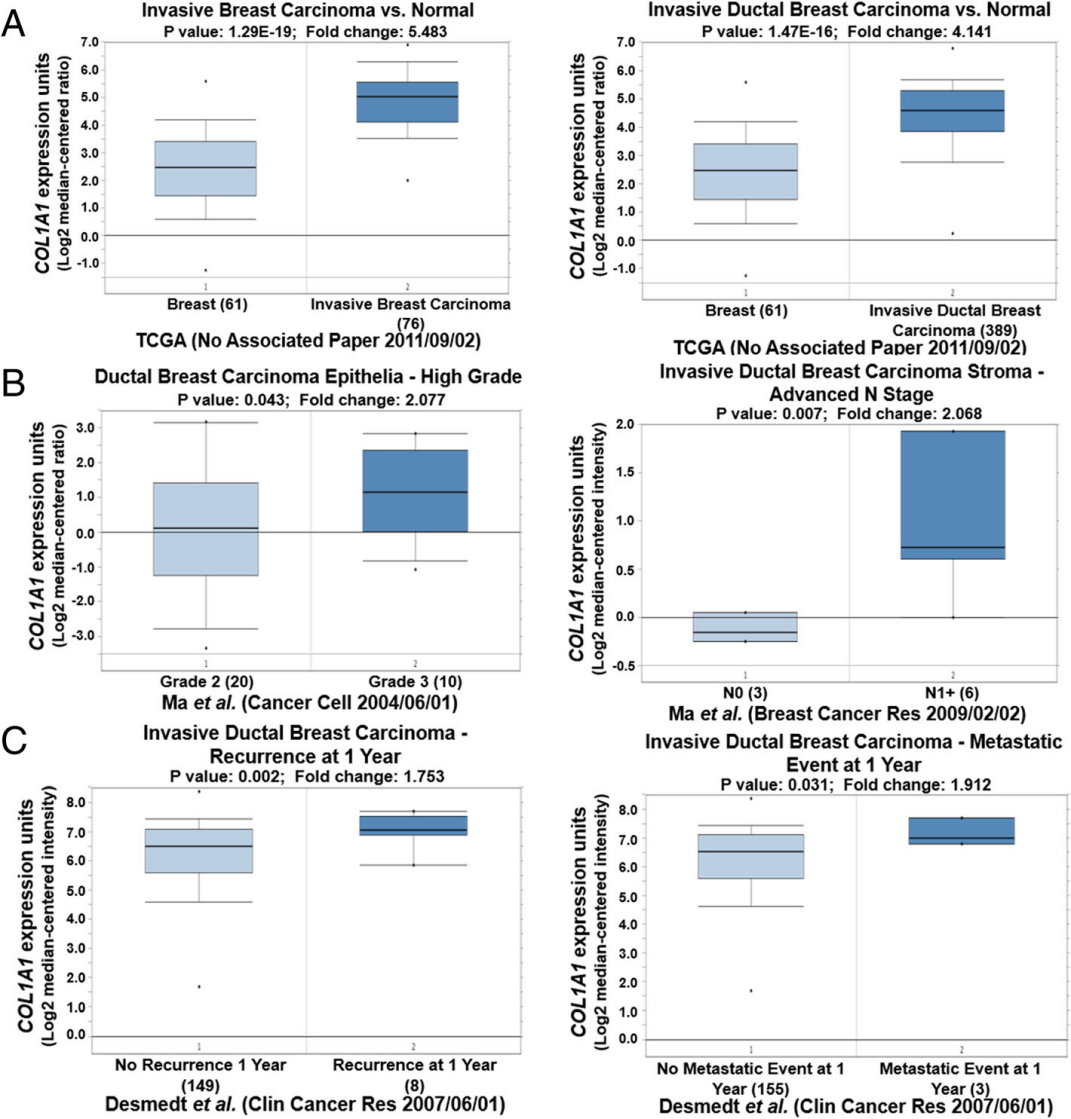
Validation of *COL1A1* expression in breast cancer using Oncomine datasets. **a** – High *COL1A1* expression was observed in breast cancer compared with normal breast tissue. **b** – High *COL1A1* expression was observed in high-grade or advanced-stage breast cancer compared with low-grade or –stage breast cancer. **c** – High *COL1A1* expression was observed in breast cancer with recurrence or a metastatic event at 1 year

We used Oncomine to investigate the expressions of *COL1A1* and *FN1* in human breast tumor invasion and metastasis. *COL1A1* was significantly increased in breast cancer compared with normal breast tissue (Fig. 4a). Furthermore, we observed a significant increase of *COL1A1* expression level in high grade and advanced N1+ stage compared with the control group (Fig. 4b). Subsequently, we studied the association between the *COL1A1* expression level and clinical outcome. A high expression level of *COL1A1* was observed in breast cancer with recurrence or a metastatic event after 1 year (Fig. 4c).

**Fig. 5:**
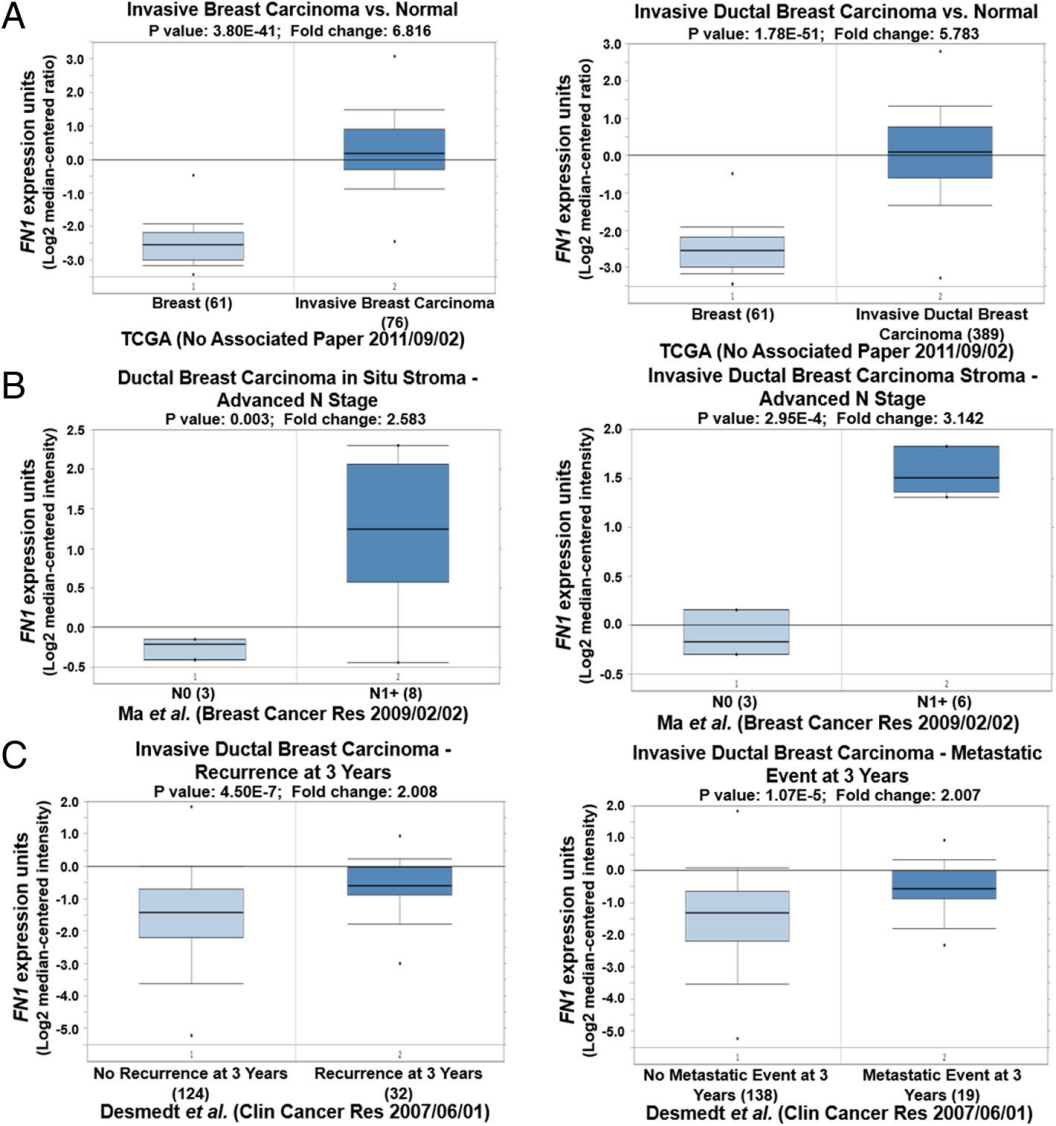
Validation of *FN1* expression in breast cancer using Oncomine datasets. **a** – High *FN1* expression was observed in breast cancer compared with normal breast tissue. **b** – High *FN1* expression was observed in high-grade or advanced-stage breast cancer. **c** – High *FN1* expression was observed in breast cancer with recurrence or a metastatic event at 3 years

Similarly to *COL1A1*, *FN1* was also highly expressed in breast cancer (Fig. 5a) and the advanced N1+ stage of breast cancer (Fig. 5b) compared with their control groups. An elevated *FN1* level in breast cancer patients with recurrence or a metastatic event was observed in comparison with controls (Fig. 5c).

These high expression levels of *COL1A1* and *FN1* were correlated to late stage of breast cancer and poor clinical outcomes.

## Discussion

Tumor angiogenesis and destruction of the extracellular matrix (ECM) are two essential factors for tumor invasion and metastasis. Previous studies concentrated on the tumor characteristics but there has been a lack of study of the tumor microenvironment. Genes altered in the tumor microenvironment may play important roles in the destruction process and be further involved in tumor progression.

To contribute to the understanding of tumor stroma roles in breast progression, we compared transcriptome profiles between cancer stroma (stroma surrounding invasive breast primary tumors) and normal breast stroma. A total of 3708 differentially expressed genes (DEGs) were identified in the GSE26910 dataset, whereas 665 DEGs were found in GSE10797. By overlapping the sets, 146 common DEGs were found.

Among the common DEGs, genes encoding matrix components were significantly dysregulated, including *MMP1*, *POSTN*, *SPARC*, *COL1A1*, *COL5A1*, *COL5A2*, *COL10A1* and *COL11A1*. These altered genes were significantly associated with the pathways of “extracellular matrix organization”, “ECM-receptor interaction” and “focal adhesion”. Our findings indicated that these genes and pathways are related to the establishment of the tumor microenvironment.

The degradation of the basal membrane and the ECM results from proteolysis. Most members of the proteolytic system are mediated by AP-1 transcription factors through binding to TPA-responsive elements (TREs) in the promoter or enhancer region of the target genes [35]. The AP-1 factor is a heterodimer consisted of proteins belonging to the Fos, Jun, ATF and JDP families [39]. In our PPI network analysis of these common genes, *JUN*, *FOS* and *ATF3* were recognized as key nodes. This suggests that they may play a critical role in tumor environment via AP-1 factor.

JUN/c-Jun is a member of the Jun family, FOS/c-Fos is a member of the Fos family and ATF3 belongs to the ATF family. AP-1 regulates several cytological processes, including differentiation, cell death, proliferation, oncogenic transformation, apoptosis and cell migration [40]. Due to its constitutive activation and the diverse responses it induces, AP-1 plays a crucial role in promoting tumor invasion and migration [41].

In our study, JUN, FOS and ATF3 were involved in 12 of the top 50 significant biological processes. These included “negative regulation of cellular process” and “negative regulation of biological process”.

*FOS* and *ATF3*, *STAT1*, *COL1A1* and *FN1* were also identified as key nodes. STAT1 mediates both type I (alpha and beta) and type II (gamma) IFNs that are associated with cell growth, regulation, and antiviral and immune defense [36]. Stromal STAT1 expression promotes tumor progression in breast cancer. COL1A1 and FN1 are individual ECM genes and have been reported to be associated with tumor invasion and metastasis. Increased extracellular levels of COL1A1 promote tumor cell invasiveness in culture and metastasis in animal models [37]. Furthermore, a high level of COL1A1 increases the likelihood of clinical metastasis of multiple human solid tumors [42, 43]. The elevated expression of *FN1* promotes lymph node metastasis in human oral squamous cell carcinoma by promoting vascular endothelial growth factor-C expression and the epithelial–mesenchymal transition (EMT) [38]. However, no study on *COL1A1* and *FN1* associations with breast cancer has been reported. Therefore, we performed Oncomine analysis to investigate their expression in breast cancer. High expression levels of *COL1A1* and *FN1* were associated with high grade or advanced stage of breast cancer and poor prognosis.

In summary, breast cancer stroma-related genes, including *JUN*, *FOS*, *ATF3*, *STAT1*, *COL1A1* and *FN1*, were all associated with tumor invasion and metastasis. This reveals that cancer stroma-related genes have important roles in tumor progression.

Despite the improvement of tumor detection tools (including imaging techniques like MRI and PET; and analysis of blood biomarkers shed by the tumor, such as proteins and cell free nucleic acids) and tumor therapies, there are still limitations. Clinical detection of tumors is limited to masses 1 cm in diameter. Resistance to therapy has been one of the major challenges in treating cancer [44].

Tumor microenvironment has been considered as prospective breakthrough point in early tumor detection and tumor treatment recently. Based on the two hallmarks of the tumor microenvironment, acidity and low oxygenation, the macromolecular near-infrared poly (ethylene glycol)-conjugatediridium (iii) complex is designed to successively respond to acidity and hypoxia while amplifying detection sensitivity via signal propagation. Primary tumors and metastasis tumor nodules as small as 1 mm in mice have been successfully detected [45]. In addition, tumor microenvironment biomarkers contribute to tumor detection and treatment. MMP-9, a major regulator inducing ECM component degradation, is highly expressed in many human cancer types compared with normal controls. Therefore, an exogenously administered tumor-penetrating nanosensor with an MMP-9 peptide substrate in its surface has been created [44]. Importantly, this nanosensor is reported to detect nodules with median diameters smaller than 2 mm in an orthotopic model of ovarian cancer. In terms of drug resistance, different components of the microenvironment have been found to participate in the development of chemoresistance and inhibit MMPs, although chemokine signaling is effective when combined with traditional chemotherapies [46].

In this study, *COL1A1* and *FN1* were all ECM genes with elevated mRNA levels in tumor stromal cells. Therefore, they might be used as key candidate genes for tumor detection and treatment.

The strength of our study is that we combined gene expression data of stroma surrounding invasive breast primary tumors and normal stroma from two GEO databases and obtained conserved genes. It is known that as breast tumors progress from ductal carcinoma in situ (DCIS) to invasive ductal carcinoma (IDC), the ECM undergoes increased collagen fiber linearization and thickening due to deposition and cross-linking of the collagen. In IDC, the collagen fibers are aligned perpendicularly to the tumor boundary, forming migration tracks for invasive tumor cells to exit the tumor tissue and enter the bloodstream [2]. Thus, the alteration of stroma composition from DCIS to IDC is important for tumor invasion. Further study may focus on the gene expression of cancer stroma in this process.

Our study also has several limitations. First, the analysis of more similar or larger databases may be necessary for identification of the conservative key genes. Second, experimental validation of the expressions of these conserved genes is needed. Third, investigation the roles of these critical genes should be conducted in the future.

## Conclusions

Our results show several key genes in breast cancer stroma, including *JUN*, *FOS*, *ATF3*, *STAT1*, *COL1A1* and *FN1*. These genes were involved in “extracellular matrix organization”, “ECM-receptor interaction” and “focal adhesion”. Oncomine analyses of *COL1A1* and *FN1* in different human breast cancer datasets suggest that these two genes may be promising targets for the future studies.

## Abbreviations

BH: Benjamini and Hochberg’s
DEGs: Differentially expressed genes
ECM: Extracellular matrix
FDR: False discovery rate
GEO: Gene Expression Omnibus
GO: Gene ontology
HPRD: Human Protein Reference Database
KEGG: Kyoto Encyclopedia of Genes and Genomes
PIP: Protein-protein interaction prediction database
PPI: Protein–protein interaction
